# Multi-omics spatial characteristics of CD8^+^TRM cells in hepatocellular carcinoma and immunotherapy response prediction

**DOI:** 10.3389/fimmu.2025.1710741

**Published:** 2025-12-08

**Authors:** Shipeng Li, Ju Ma, Xinqiang Li, Bingbing Qiao, Liancai Wang, Deyu Li, Hua Zhou, Haixu Xu, Jinzhen Cai

**Affiliations:** 1 Department of Hepatopancreaticobiliary Surgery, Henan Provincial People’s Hospital, Zhengzhou University, Zhengzhou, China; 2 Organ Transplantation Center, Affiliated Hospital of Qingdao University, Qingdao, China; 3 Department of Hepatopancreaticobiliary Surgery, First Affiliated Hospital of Zhengzhou University, Zhengzhou, China; 4 Endocrinology and Metabolism Center, Henan Key Laboratory of Rare Diseases, The First Affiliated Hospital, and College of Clinical Medicine of Henan University of Science and Technology, Luoyang, China; 5 Department of Immunology, Key Laboratory of Immune Microenvironment and Disease of the Educational Ministry of China, Tianjin Key Laboratory of Cellular and Molecular Immunology, School of Basic Medical Sciences, Tianjin Medical University, Tianjin, China; 6 Organ Transplant Center, Fujian Medical University Union Hospital, Fuzhou, China

**Keywords:** hepatocellular carcinoma, immunotherapy, CD8+TRM cells, immune checkpoint blockade, tumor-infiltrating immune cells, tissue-resident memory

## Abstract

**Introduction:**

Understanding CD8^+^ tissue-resident memory T cells (TRM) spatial characteristics in hepatocellular carcinoma (HCC) is challenging, and clarifying the spatial feature changes following immunotherapy represents an urgent research gap.

**Methods:**

This study employs a multi-omics approach to analyze the spatial distribution and intercellular interactions of TRM cells in HCC tissues using radiomics, single-cell sequencing, and multiplex immunofluorescence histochemistry (m-IHC).

**Results:**

Our results show that the number of CD8^+^ TRM cells in HCC increases following immunotherapy. Furthermore, after dividing tumor tissues into the tumor core (TC), invasion margin (IM), and normal tissue (N), a increase in CD8^+^ TRM cells from the IM to the TC can be observed. Consistent with the results of single-cell sequencing analysis, this change in spatial characteristics may be associated with the interactions between CD8^+^ TRM cells and CD68^+^ cells.

**Discussion:**

Immunotherapy can modify the spatial characteristics of CD8^+^ TRM cells via regulating their crosstalk with other immune cells, and the spatial distribution of CD8^+^ TRM cells in the HCC tumor microenvironment (TME) correlates with immune checkpoint blockade (ICB) therapeutic efficacy. Clarifying the mechanisms of action of immunotherapeutic drugs and developing a non-invasive radiomics model to predict CD8⁺ TRM cell dynamics will facilitate the clinical management of HCC.

## 1 Introduction

Hepatocellular carcinoma, the most prevalent primary hepatic malignancy, ranks among the commonest primary tumors of the gastrointestinal-digestive system worldwide, whose rates of morbidity and mortality continue to increase globally (1, 2), and systematic antitumor therapy represents the main option for treating patients with mid-advanced HCC (3, 4). Immune checkpoint blockade involves the use of antibodies to target specific molecules. Some examples include anti-PD-1/PD-L1 and anti-CTLA4 inhibitors, which have been shown to be effective for treating melanoma, non-small cell lung cancer (NSCLC), renal cell carcinoma (RCC), breast carcinoma (BC) as well as other cancers (5, 6). The most widely used ICBs are the first-generation immune checkpoint inhibitors targeting PD-1 and PD-L1 (7), which can block tumor-related downregulation of the immune system to enhance antitumor immunity and offer a broad anticancer spectrum (8, 9). ICB represents a particularly promising treatment for advanced HCC due to the abundant immune cell infiltration in HCC tumor microenvironment (10, 11). The tumor microenvironment (TME) of HCC is comprised of infiltrating stromal and immune cells. These cells confer HCC immunogenicity and immunosuppressive characteristics, producing an upregulation of immune checkpoint molecules on their surface (12). Nivolumab and Pembrolizumab are the two main inhibitors of PD-1, while the PD-L1 inhibitor, Atezolizumab, is widely used clinically. As we know, when PD-1 and PD-L1 are bound, T cell activity is inhibited, and these drugs exert their functional activity by blocking the binding of PD-1 to PD-L1, thus triggering T cells to re-recognize and attack tumor cells (13).

The most extensively studied biomarkers for anti-PD-1/PD-L1 therapy include tumor mutation burden (TMB), expression of PD-L1 in tumor/immune cells and expression patterns of immune-related genes in the tumor microenvironment (14–16). Interestingly, tumor-infiltrating immune cells (TIICs) are closely related to ICB efficacy due to their heterogeneity and exhibition of functional and phenotypic plasticity (17, 18). As a key member of TIICs, tissue-resident memory (TRM) cells operate as front-line adaptive immune sentinels within the liver and exert crucial immune surveillance functions (19), they involve a resilient, non-recirculating memory T-cell lineages that reside in tissues and are classically defined by expression of the tissue retention markers, CD69, CD103 and CD49a (20–22). The CD8^+^TRM cells exhibit a unique aptitude for development and long-term persistence within tumors and their associated microenvironments, a trait that allows them to sustain their effector activity and mount a response to ICB therapy. Regarding the correlation between TRM cells and ICB treatment efficacy, studies have reported that the number of CD8+TRM cells is correlated with the tumor microenvironment and the efficacy of immunotherapy in patients with NSCLC (23); the TRM cells from HBV-related HCC express higher levels of PD-1 are functionally more suppressive and exhausted than those from non-virus-related HCC and respond poorly to immune checkpoint blockade (24). However, few reports are available on the spatiotemporal changes of TRM distribution in HCC patients after anti-PD-1/PD-L1 treatment.

Herein, we initially predicted the distribution changes of CD8^+^ T cells after ICB treatment by constructing a radiomics model. Then using single-cell RNA sequencing within tumor tissues of HCC patients and mIHC, we found that the proportions of CD8^+^T cells and CD8^+^TRM cells generally increased while that of CD4^+^T cells significantly decreased following ICB treatment. Moreover, infiltration of CD8^+^TRM cells increased from IM to TC in the ICB-treating group which might be related to the occurrence of crosstalk between CD68^+^ cells and CD8^+^TRM cells. Our data provide important new information to support the evaluation of CD8^+^TRM cell density and spatial patterning amidst anti-PD-1/PD-L1 treatment/PD-L1DATA that is pivotal for advancing our understanding of the determinants governing immunotherapy response outcomes in HCC patients and provides feasibility for the subsequent non-invasive prediction of the efficacy of ICB therapy in HCC.

## 2 Methods

### 2.1 Patients

A total of 302 patients who underwent abdominal CT scans and were pathologically diagnosed with hepatocellular carcinoma (HCC) were enrolled from Henan Provincial People’s Hospital and The First Affiliated Hospital of Zhengzhou University between 2023 and 2024. Ultimately, 50 patients meeting the predefined inclusion criteria were enrolled for radiomics model construction. Inclusion Criteria: Histopathologically confirmed hepatocellular carcinoma (HCC) (diagnosis based on WHO 2019 criteria); Preoperative contrast-enhanced computed tomography (CT) of the liver performed within 2 weeks prior to surgery, with images covering the entire tumor; Either received standard immunotherapy or had no history of prior anticancer treatment (such as chemotherapy, targeted therapy, local ablation). Exclusion Criteria: Suboptimal imaging quality (severe motion artifacts) or indistinct tumor boundaries that precluded accurate segmentation; Incomplete clinical or pathological data (such as missing tumor stage, histological grade); A history of other primary malignant tumors (except for cured non-melanoma skin cancer or *in situ* cervical cancer).

Then after excluding patients who with concurrent autoimmune diseases, HIV, or syphilis; with severe diseases of brain, heart, kidney and other organs; with no follow-up information after surgery; cannot tolerate immunotherapy or have obvious complications, 12 of them were selected (**Supplementary Figure S1**), among the 12 patients, 6 received regular anti-PD-1/PD-L1 antibodies (Sintilimab/Tislelizumab/Atezolizumab) therapy (Administered once every 3 weeks, with a total of more than 3 treatment cycles) before surgery were divided into the ICB-treating group(EG), another 6 patients were treatment-naïve prior to the surgery were untreated group(NG). The demographic and clinicopathologic characteristics of the study participants are presented in **Table 1**. This research was conducted in compliance with the Declaration of Helsinki and received prior approval from the Ethics Committee of Henan Provincial People’s Hospital. Written informed consent was obtained from all participants or their legal guardians before enrollment.

**Table 1:** Characteristic information of HCC patients with immunotherapy.

Characteristic	Total N=12	NG group N=6	EG group N=6
Age (years)	58.5 (56-64)	63 (56-70)	58 (56-59)
≥65years	3 (25%)	3 (50%)	0 (0%)
Sex (male/female)			
Male	10 (83%)	5 (83%)	5 (83%)
Female	2 (17%)	1 (17%)	1 (17%)
α-fetoprotein			
≥400 µg/l	4 (33%)	3 (50%)	1 (17%)
< 400 µg/l	8 (67%)	3 (50%)	5 (83%)
Child-Pugh score			
5-6	11 (91.7%)	5 (83%)	6 (100%)
7-9	1 (8.3%)	1 (17%)	0 (0%)
Hepatitis B virus status			
Positive	11 (91.7%)	5 (83%)	6 (100%)
Negative	1 (8.3%)	1 (17%)	0 (0%)
Barcelona clinical liver cancer stage			
A	11 (91.7%)	5 (83%)	6 (100%)
B	1 (8.3%)	1 (17%)	0 (0%)

Data are median (IQR) or n (%).

### 2.2 CT examinations

All patients underwent contrast-enhanced computed tomography (CE-CT) of the liver. CT images were acquired in the axial plane with a slice thickness of 1–5 mm using 64-section, 128-section, or 256-section multidetector CT (MDCT) scanners (GE Revolution; GE Healthcare, Milwaukee, WI, USA). Scanning parameters were standardized where possible: tube voltage was set to 120 kVp, and tube current was adjusted using automatic exposure control (AEC) technology to balance image quality and radiation dose.

### 2.3 Samples collection and examination

All cases were histopathologically confirmed HCC by clinical history and histologically reviewed by liver pathologists. To mitigate sample degradation, tissue specimens were harvested promptly upon excision from the surgical field. Tumor tissues were carefully dissected, rinsed with sterile phosphate-buffered saline (PBS) to remove blood and debris, and then bisected along their longitudinal axis. Tissues designated for research were subsequently collected from the freshly exposed tumor surface using a sterile scalpel, ensuring minimal damage to the tissue microstructure, every sample was about 2cm long and 1cm wide to ensure that each of it contained well-defined tumor center, borderline areas, and normal surrounding tissue. All specimens were soaked in formalin and preserved at room temperature before further application. Laboratory examination data included aspartate transaminase (AST), Albumin (ALB) and total bilirubin (TBiL) were assayed by the clinical laboratory of Henan Province People’s Hospital and First Affiliated Hospital of Zhengzhou University.

### 2.4 Immunohistochemistry

Formalin-fixed, paraffin-embedded (FFPE) tissues were sectioned at 4 μm thickness, mounted on adhesive slides, and incubated at 60 °C for 30 minutes to enhance tissue adhesion. Sequential processing included deparaffinization in xylene, rehydration through graded ethanol series, and endogenous peroxidase blockade with 3% hydrogen peroxide, and closed antibody incubation for 30 min. Next, incubated with primary antibodies of CD8, CD4 and CD68 for night. Then, incubated with second antibody enhance kits for 60 min, and tissue color staining with DAB under a microscope.

### 2.5 Immunohistochemical evaluation

**Figure 1:**
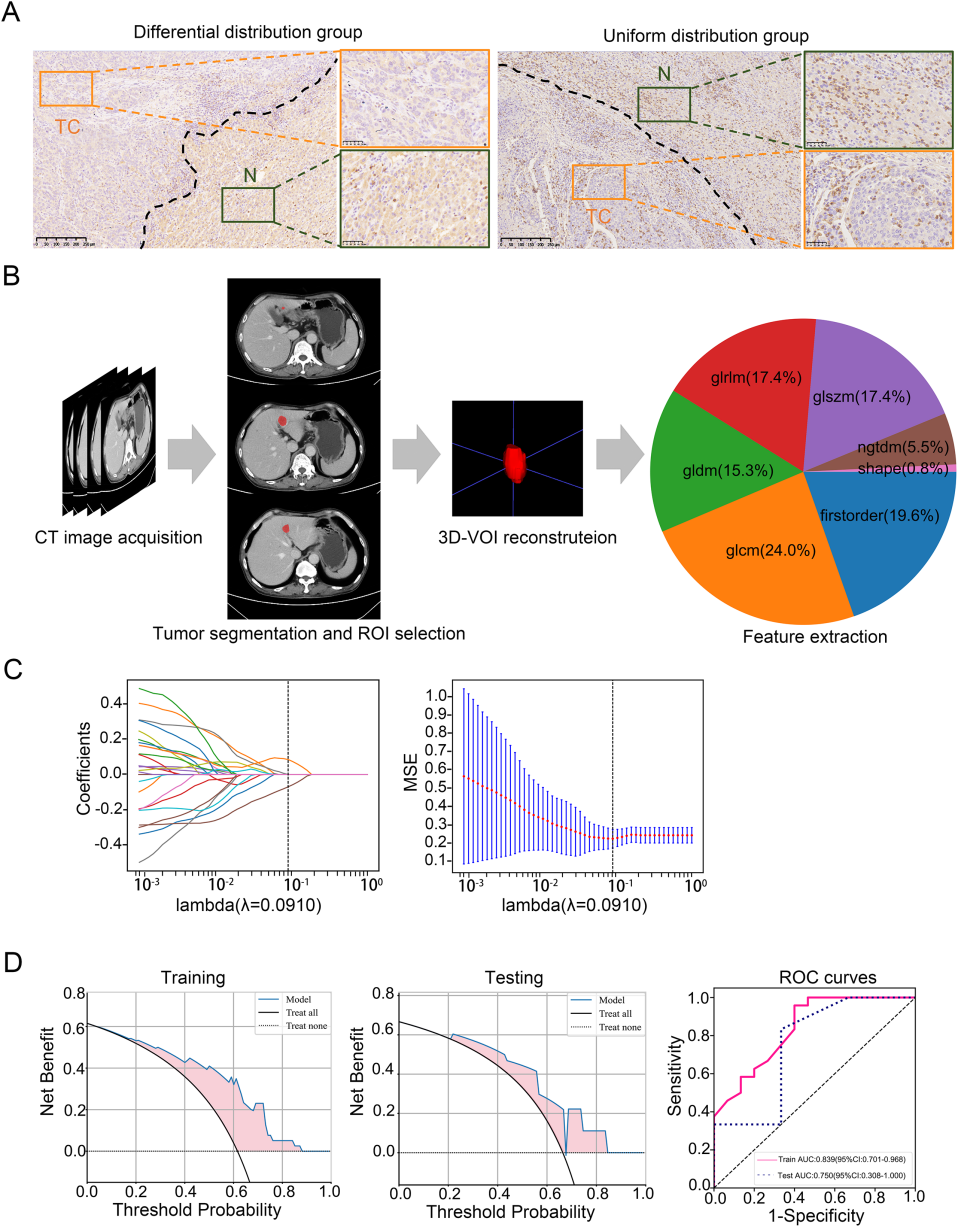
Workflow of model construction and validation. **(A)** Representative CD8 immunohistochemistry staining images of different groups. Scale bar: 500μm, 100μm **(B)** ROI delineation and radiomics feature extraction: The largest tumor region in the contrast-enhanced CT arterial phase was segmented slice-by-slice as ROI; 1834 features were acquired via the radiomics extension module of 3D Slicer. **(C)** Optimal radiomics features were selected via LASSO regression combined with 5-fold cross-validation. **(D)** Diagnostic performance of the radiomics-based predictive model was evaluated using decision curve analysis (DCA) and area under the ROC curve (AUC) in the training and validation cohorts. TC, tumor core; N, normal tissue.

ImageJ software, an open-source image analysis tool, was downloaded from the official website (https://imagej.nih.gov/ij/), the IHC tool box plugin for ImageJ was installed according to the instructions provided by the plugin developers, and the plugin automatically detects stained regions based on color thresholds. These thresholds are adjusted according to the staining intensity and background color of each individual image. Subsequently, the plugin calculates the percentage of positive staining area, average staining intensity, and other relevant parameters for each image (25). After evaluating the immunohistochemical scores of tumor tissues and adjacent normal tissues, those with significant score differences were divided into differential distribution group (**Figure 1A**).

### 2.6 Texture feature analysis image segmentation

Tumor segmentation was performed by two radiologists, blinded to clinicopathologic data, using ITK-SNAP (v4.2.0; https://www.itksnap.org). Regions of interest (ROIs) were manually delineated slice-by-slice on CT images, and fused to generate a 3D volume of interest (VOI; **Figure 1B**). Contouring criteria: (1) trace tumor margins; (2) include intratumoral necrosis/hemorrhage; (3) exclude adjacent organs. All segmentations were confirmed by a senior radiologist, with disagreements resolved via consensus.

### 2.7 Feature extraction and selection

Radiomics features (n=1,834 texture-based) were extracted via the Artificial Intelligent Kit (v3.2.2), including first-order (n=359), shape (n=15), GLCM (Gray Level Co-occurrence Matrix, n=440), GLRLM (Gray Level Run Length Matrix, n=319), GLSZM (Gray Level Size Zone Matrix, n=319), GLDM (Gray Level Dependence Matrix, n=281), and NGTDM (n=101) features. Patients were first divided into differential/uniform distribution groups, then randomly assigned to training/validation cohorts (4:1).

Data preprocessing: Missing values were imputed with feature-column medians, followed by Z-Score normalization. Redundant features were removed via ANOVA-KW (p > 0.05 excluded) and correlation analysis (|r| > 0.8 filtered). LASSO regression with 5-fold cross-validation was used for dimensionality reduction, retaining 2 optimal features (1 GLCM, 1 GLSZM) to construct the radiomics signature. The workflow is shown in **Figures 1B, C**.

### 2.8 Multi-spectral imaging

Multi-spectral images were visualized in Phenochart (v1.1.0). Regions of interest (ROIs) were defined as: normal (N, tumor-free area in the specimen), invasion margin (IM, ~1–1.5 mm-wide tumor-normal interface, depth matched to microscope field), and tumor (TC, tumor core). Fixed-size ROIs (931 × 698 μm; 20× objective) were selected via Phenochart’s stamping tool, guided by pre-acquired whole-slide scans. Viable regions were prioritized for selection (maximized count, minimal overlap per specimen). All data underwent quality control (QC): inappropriate regions were excluded, and outliers were confirmed. Results were interpreted by a pathologist with 10 years of experience.

### 2.9 Multiplex immunohistochemistry

mIHC was performed to visualize the expression of CD8, CD4, CD69, CD68, PD-1, and CD103 (**Supplementary Table S1**). Paraffin sections were deparaffinized in xylene and rehydrated in a graded ethanol series. Heat-induced antigen retrieval was performed in EDTA buffer (pH 9.0) using a microwave. Sections were blocked with commercial blocking buffer for 10 min. Antibody concentrations and staining order were pre-optimized; slides were incubated sequentially with primary antibodies and HRP-conjugated secondary antibodies. After each TSA round, heat-mediated antibody stripping was done to avoid cross-reactivity. Finally, nuclei were counterstained with DAPI.

### 2.10 Recognition of cell morphology and spatial distribution

The feature extraction of multispectral images was performed using the Image J The characteristics of the cell phenotype. We analyzed the proportion and spatial distribution of each cell type in the tumor microenvironment (TME). Within a 30 μm radius, central immune cells were paired with surrounding cells to construct spatial interaction networks, which were quantified using two indicators:

Effective score: Number of target neighboring cells/Number of central immune cells (reflects average target cells per central immune cell).

Average distance: Sum of distances between central cells and target neighboring cells/Number of target neighboring cells.

Since ligand-receptor interactions (mediating anti-tumor/immunosuppressive effects) require cell proximity, these spatial metrics aid in interpreting cell functions in the TME.

### 2.11 Single cell gene expression quantification and subcluster delineation

Raw and processed data were downloaded from the GEO database (https://www.ncbi.nlm.nih.gov/geo/), specifically GSE151530 (26), GSE138709 (27) and GSE156625 (28). Following the methodology outlined in our previous studies (29–32), the Seurat R package (version 4.3.0) was used for data import and processing, which included quality control (QC), normalization, and scaling (33). scRNA-seq data were filtered to exclude cells with <501 expressed genes or >25% mitochondrial gene counts. Normalization and scaling were performed with default Seurat parameters. Highly variable genes were identified using FindVariableFeatures. PCA was conducted on variable genes; cell clustering was done via FindNeighbors (dims = 1:10) and FindClusters (resolution = 0.5). tSNE and UMAP were used for non-linear visualization.

### 2.12 Cell type determination

Distinctive features within each cluster were identified using the FindMarkers and FindAllMarkers functions. To assign cell types to known biological types, we referenced the CellMarker database (https://bio-bigdata.hrbmu.edu.cn/CellMarker/) and relevant published articles, utilizing canonical marker genes. Additionally, the SingleR R package (version 2.0.0) was employed to further confirm cell type assignments (34).

### 2.13 Functional enrichment analysis

Post cell type annotation, functional enrichment analysis was performed on differentially expressed genes (DEGs) across clusters. Gene Ontology (GO, focusing on Biological Processes) and KEGG pathway analyses were conducted to characterize the biological functions of each cell type. We standardized the enrichment analysis workflow using clusterProfiler (Version 4.6.2) (35) for term enrichment calculation and org.Hs.eg.db (Version 3.16.0) for accurate human gene ID mapping. Both GO and KEGG analyses adopted a consistent statistical significance cutoff of p < 0.05. The results were summarized by selecting the top 10 enriched terms, which were visually displayed via barplots or dotplots.

### 2.14 ssGSEAscore survival curve analysis

Gene expression profiles and clinical annotations of hepatocellular carcinoma (LIHC) patients were downloaded from The Cancer Genome Atlas (TCGA) database (https://portal.gdc.cancer.gov/). After quality control and normalization, a curated list of immune-related gene sets (CD8^+^T: CD3D CD8A; CD8^+^TRM: CD3D CD8A CD69 ITGAE CXCR6 RUNX3), the GSVA R package was employed to compute ssGSEAscores for each sample, and samples were divided into high and low ssGSEAscore groups using the X-tile software to identify the optimal cutoff value maximizing survival differences.

### 2.15 Cell-cell communication analysis

Cell-to-cell interactions were inferred using CellChat (v1.6.1) (36). Ligand-receptor (L-R) pairs were annotated based on the KEGG database and recent experimental literature. The analysis included three core steps: (1) identifying signaling-related differentially expressed genes, (2) computing ensemble average expression, and (3) calculating intercellular communication probability.

### 2.16 Statistical analysis

All statistical analyses were performed using GraphPad Prism 9, R version 4.1.0 and SPSS version 28.0. Continuous variables were summarized as mean ± standard deviation (SD) for normally distributed data and median with interquartile range (IQR) for non-normally distributed data. Categorical variables were presented as frequencies and percentages (%). The overall treatment effect was first evaluated by comparing all treated subjects against untreated controls using Within each treatment arm, subgroup differences were assessed through one-way ANOVA (with Tukey’s *post-hoc* pairwise comparisons) or Kruskal-Wallis tests (followed by Dunn’s procedure with Bonferroni adjustment) (comparison of the proportion of CD8^+^ TRM cells between different groups), depending on data distribution and variance homogeneity. Direct comparisons between corresponding subgroups across arms were conducted using analogous parametric or non-parametric tests. A two-way ANOVA model testing the treatment-by-subgroup interaction term was employed to determine whether therapeutic effects differentially manifested across subgroup categories. All correlation analyses were performed using Pearson’s method to calculate the correlation coefficient (correlation of immune cell counts). Statistical analysis and visualization were performed using. All P values were two-tailed, and P < 0.05 was used to define statistical significance.

## 3 Results

### 3.1 Patient features in radiomics model

Based on the inclusion and exclusion criteria, 50 HCC patients were included in the radiomics analysis. The cohort had a mean age of 57.22 years (range: 34–86 years), with a male predominance (33/50, 66.0%). The majority of patients were HBsAg-positive (39/50, 78.0%).

Patients were randomly split into the training cohort (n = 40) and validation cohort (n = 10) at a 4:1 ratio. As shown in **Table 2**, the two cohorts were well-balanced in terms of demographic features and clinicopathologic characteristics (including HBsAg status, tumor stage, etc.), with no statistically significant differences observed (all p > 0.05).

**Table 2:** The clinical-pathologic characteristics of patients.

Characteristic	Training group(n=40)	Testing group(n=10)	P value
Age, year (mean ± SD)	56.93 ± 10.75	58.4 ± 9.51	0.694
Sex (Male/Female)	27/13	6/4	0.65
ALB, g/L (mean ± SD) ALT, U/L (median, IQR) AST, U/L (median, IQR) AFP, ng/mL (>200/≤200) HBsAg (positive/negative)	39.99 ± 4.77 31.93(17.85-32.88) 29.80(18.95-33.35) 9/31 32/8	37.81 ± 2.85 36.87(16.90-35.95) 35.35(23.43-35.08) 2/8 7/3	0.175 0.724 0.487 0.86* 0.49*

SD, Standard Deviation; QR, Interquartile range; ALT, Alanine transaminase; AST, Aspartate transaminase; AFP, Alpha fetoprotein; *Yates’s correction for continuity.

### 3.2 Construction of the radiomics model

In total, 1834 radiomics features were extracted from the ROIs (**Figure 1B**), after the LASSO algorithm’s feature reduction and 5-fold cross-validation (**Figure 1C**), Two features (lbp_3D_m2_glszm_Small Area High Gray Level Emphasis, wavelet_LHH_glcm_Imc2) were selected to build an Extra Trees-based radiomics model. The lbp_3D_m2_glszm_Small Area High Gray Level Emphasis is a composite index combining 3D local texture analysis with regional gray level distribution characteristics and the wavelet_LHH_glcm_Imc2 is a medical radiomic composite feature that quantifies the complexity of high-frequency texture in the vertical direction and pixel gray level co-occurrence relationships in 3D medical images. They can evaluate tumor biological behavior, treatment response, and patient prognosis by quantifying the distribution characteristics of in images (37, 38). In the training set, the model achieved an AUC of 0.893 (95% CI: 0.7102–0.9675) with sensitivity 0.917, specificity 0.600, and accuracy 0.795. In the validation set, the AUC was 0.750 (95% CI: 0.3077–1.0000) with sensitivity, specificity, and accuracy all at 0.667. Calibration curves showed good agreement between predicted and actual CD8^+^ T cell distribution (**Figure 1D**). Using the model to predict the distribution characteristics of CD8^+^T cells in HCC tissues, we found that the model predicts a positivity rate of 66.67% for the 12 patients in the Untreated group and ICB-treating group.

### 3.3 Histopathological changes of HCC after immunotherapy

**Figure 2:**
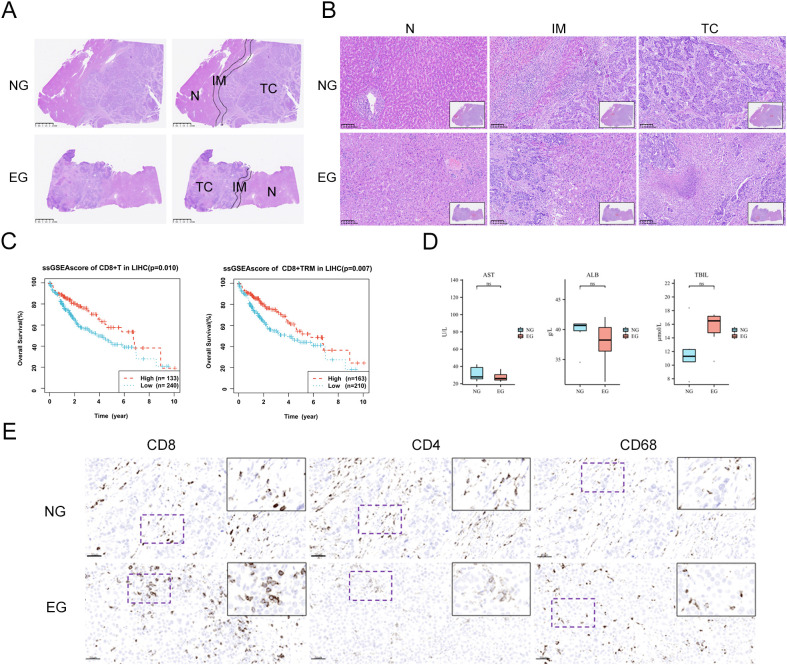
Tumor-infiltrating immune cells in HCC tissues. **(A)** Division of the TC, tumor core; IM, invasion margin; N, normal tissue. Scale bar: 2.5mm **(B)** Selection of ROIs in representative sections of H&E-stained FFPE tissues Scale bar: 100μm. **(C)** ssGSEAscore Survival Curve in LIHC of CD8^+^T (*p* = 0.010) and CD8^+^TRM (*p* = 0.007) **(D)** the difference of clinical data **(E)** IHC showed the expression of CD8, CD4 and CD68 in Untreated group (NG) and ICB-treating group (EG) respectively.

Haematoxylin and eosin(H&E)-stained tissue sections were reviewed to identify tumor core (TC), invasion margin (IM), and normal (N) areas (**Figure 2A**). ROIs were randomly selected among them (**Figure 2B**) in order to calculate the cell density and proportion in different regions. Based on ssGSEAscore Survival Curve Analysis, we can find that the infiltration levels of CD8^+^ T cells were associated with prolonged overall survival (OS) in hepatocellular carcinoma patients, likewise, CD8^+^TRM showed a similar correlation with longer OS (**Figure 2C**). By comparing imaging data before and after treatment (**Table 3**), all 6 patients who received ICB treatment in our cohort achieved partial response (PR) or stable disease (SD) as evaluated by the RECIST 1.1 criteria, demonstrating favorable therapeutic effects, and there was no significant difference of AST, ALB and TBiL levels between two groups (**Figure 2D**). Subsequently, by comparing the corresponding IHC scores, we found that following immunotherapy, the number of CD8-positive T cells in the TC was significantly increased, whereas the number of CD4-positive T cells was decreased (**Figure 2E**). This provides preliminary evidence that immune therapeutic efficacy is associated with immune cell infiltration, that it can enhance immune cell infiltration in HCC, and thereby confer a favorable prognosis to patients.

**Table 3:** Tumor volume change and reduction rate.

Items	Median	Range	Mean	SD	P value
pre-IT TS(cm)	8.7	5.2-22	10.88	6.03	0.005
Mid-IT TS(cm)	7.8	4-20	9.31	5.78	
Pre-IT TV(cm^3^)	169.9	70.4-390.6	212.85	137.78	0.003
Mid-IT TV(cm^3^)	138.1	57.6-340.7	117.97	124.70	
TVIR(%)	13.51	12.78-30.21	18.97	7.34	

SD, Standard Deviation; Pre-IT TS, Pre-Immunotherapy Tumor Size; Mid-IT TS, Mid-Immunotherapy Tumor Size; Pre-IT TV, Pre-Immunotherapy Tumor Volume; Mid-IT TV, Mid-Immunotherapy Tumor Volume; TVIR, Tumor Volume Reduction Rate.

### 3.4 Single-cell transcriptomic atlas in HCC tissues after ICB treatment

**Figure 3:**
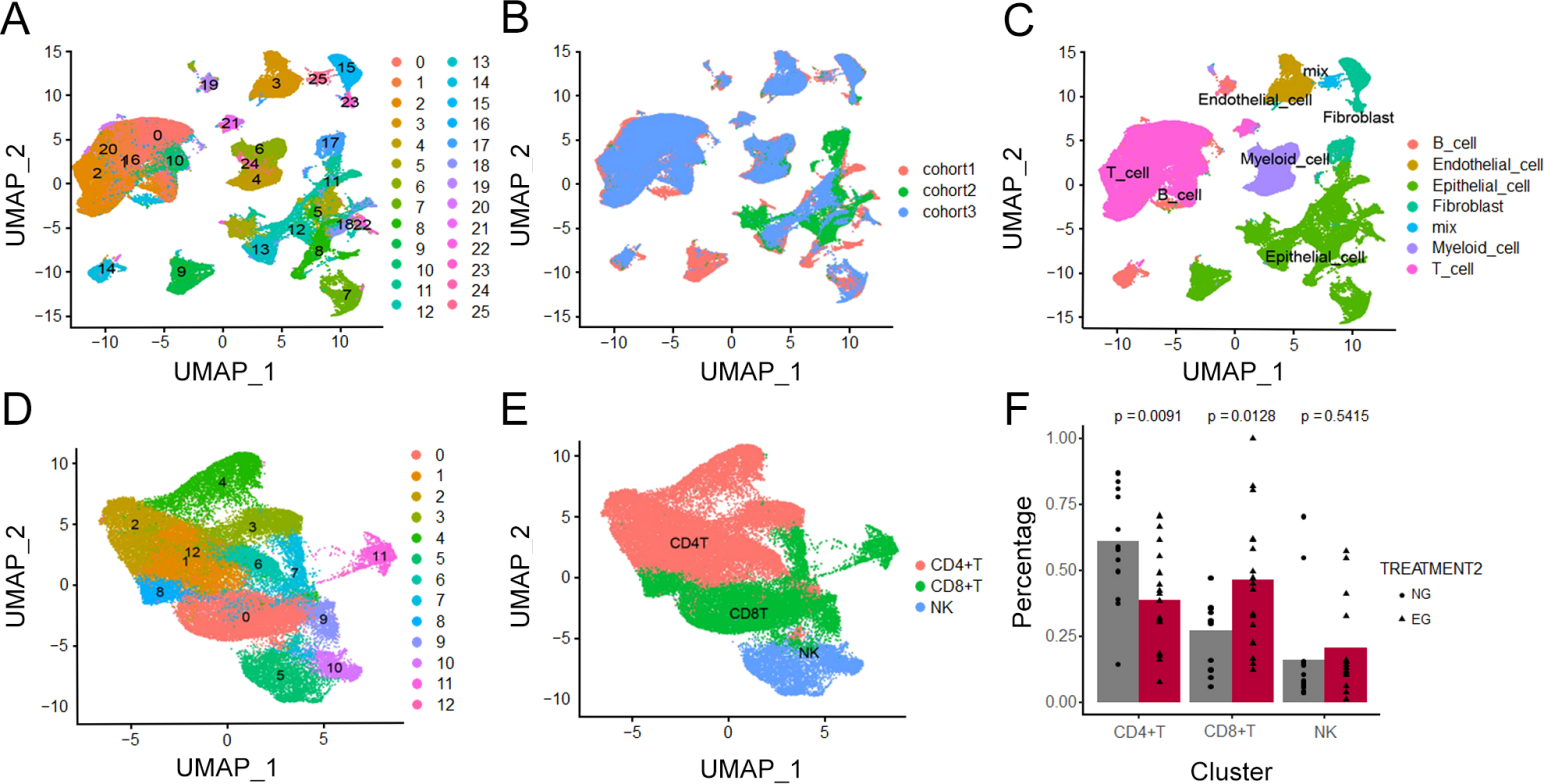
Single-cell transcriptomic atlas after ICB treatment. **(A–C)** UMAP of seven main types and their subtypes recovered from tumor tissue from patients of Untreated group (NG) and ICB-treating group (EG). Cells are color-coded by cell type accordingly **(D, E)** UMAP plots of T cell subtypes in ICB treated group **(F)** Bar graph showed percentage of CD4^+^T cells, CD8^+^T cells and NK cells of in Untreated group (NG) and ICB-treating group (EG).

To gain deeper insights into how ICB therapy reshapes the cellular composition of the HCC TME, we leveraged previously published single-cell data from HCC samples treated with ICB (**Figures 3A, B**). After quality control and doublet removal, we integrated tumor and adjacent normal tissue cells using Harmony to correct batch effects. Subsequent clustering analysis, combined with marker-based annotation, allowed us to define the major cell populations, including T/NK cells, B/plasma cells, myeloid cells, endothelial cells, epithelial cells, and fibroblasts (**Figure 3C**), considering T cells play an important role in anti-tumor immunity, we applied type-specific markers that categorized T cells into CD4^+^T, CD8^+^T and NK cells aimed to compare the percentage of different subtypes (**Figure 3D**). The proportion of CD8^+^T cell was increased in tumor tissue, but the proportion of CD4^+^T was decreased (**Figures 3E, F**).

### 3.5 CD8^+^T and CD8^+^TRM cells accumulate in tumor tissue under ICB treatment

**Figure 4:**
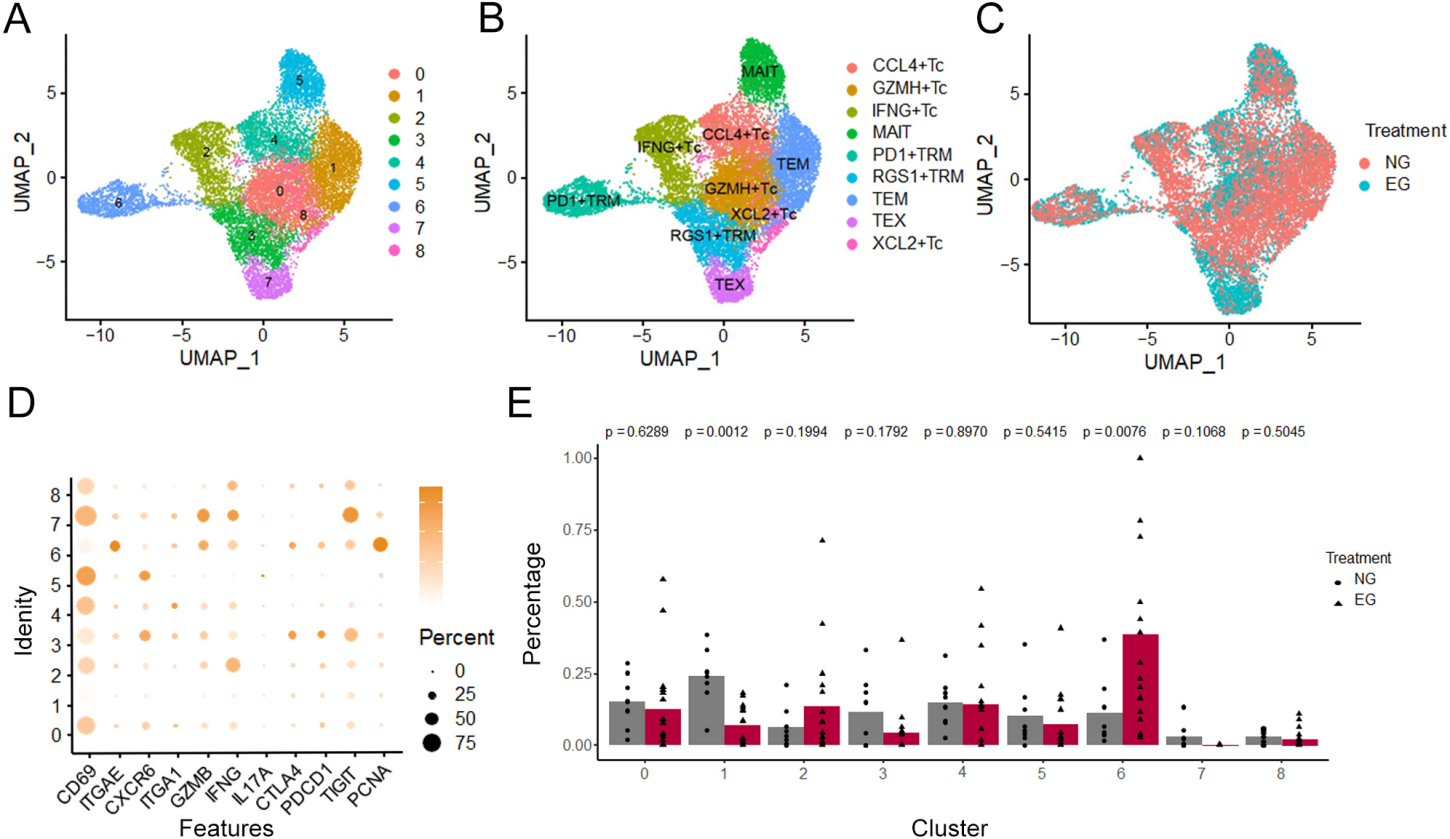
Characterization of T cells after ICB treatment. **(A–C)** UMAP plots of T cell subtypes in tumor tissues and difference in Untreated group(NG) and ICB-treating group (EG). **(D)** Dot plot showing the differentially enriched genes expression of T subtypes. **(E)** Bar graph showed percentage of T subtypes in Untreated group (NG) and ICB-treating group (EG).

To further explore the heterogeneity of CD8^+^T cell under ICB treatment, we further classified CD8^+^T in tumors into 9 subtypes (**Figure 4A**), including 4 subtypes of cytotoxic T cells (CCL4^+^Tc, GZMH^+^Tc, IFNG^+^Tc and XCL2^+^Tc), two subtypes of TRM with CD103 expression (PD-1^+^TRM, RGS1^+^TRM), mucosal-associated invariant T cells with MR1 expression, exhausted T cells (TEX) with multiple inhibitory receptors expression and effective memory T cell (**Figure 4B**). Then, we investigated the alteration of each subtype between Untreated group and ICB-treating group (**Figure 4C**), consistent with our spatial profiling results, PD-1^+^TRM cells were found to be significantly enriched in tumor parenchyma; in contrast, the abundance of other CD8^+^ T cell subtypes did not differ substantially across tissue compartments, and by comparing the gene expression of each subtype of CD8^+^T cell, we identified PCNA was enriched in the PD-1^+^TRM (**Figure 4D**), In line with the result of the increasing proportion of PD-1^+^TRM in ICB-treating group. These data suggest a potential influence of CD8^+^TRM and CD8^+^PD-1^+^TRM cells on responsiveness to ICB therapy (**Figure 4E**).

### 3.6 The interaction between CD8^+^TRM and TAMs under ICB treatment

**Figure 5:**
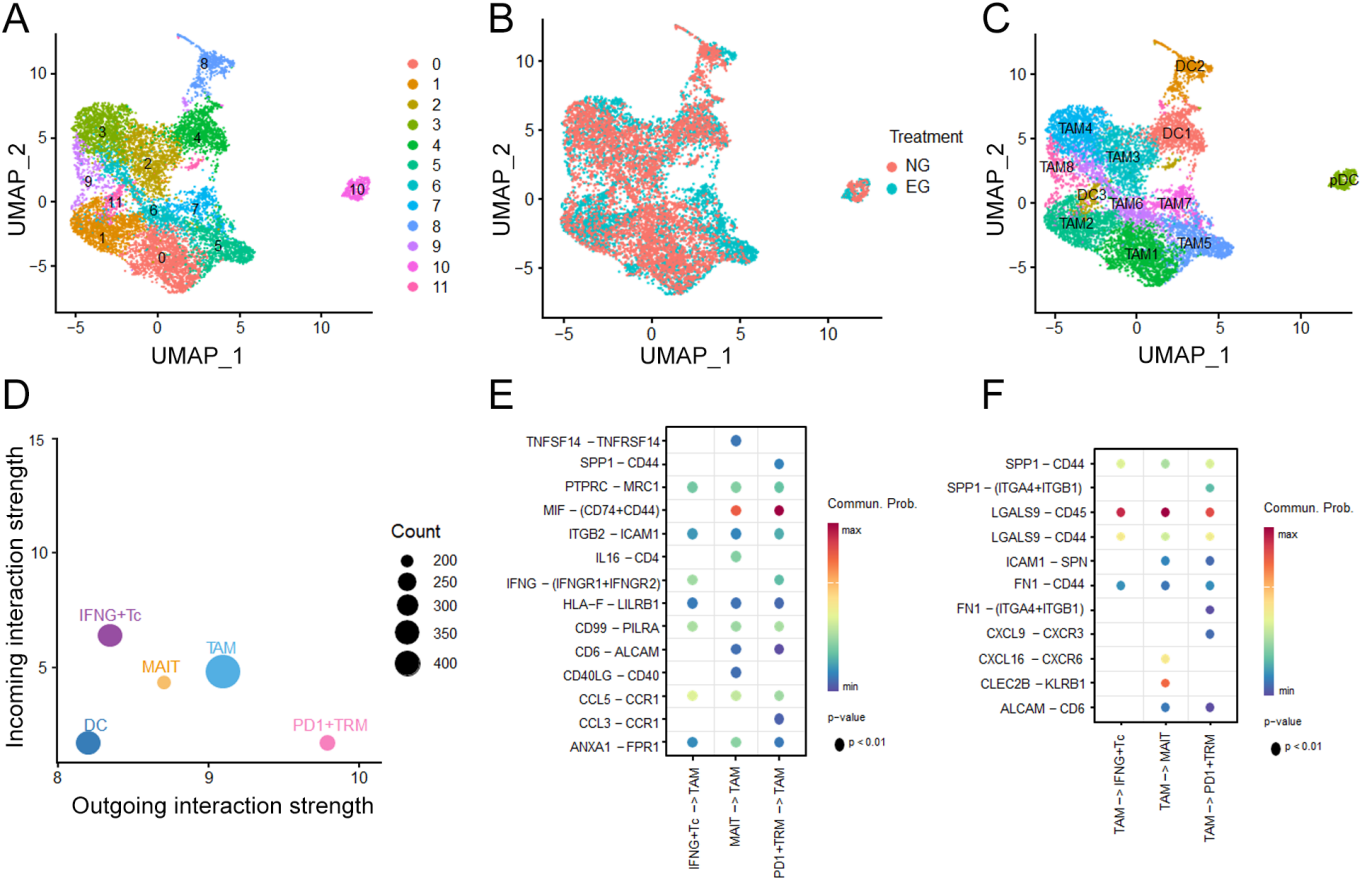
Characterization of Myeloid cell after ICB treatment. **(A)** UMAP plots of Myeloid cell clusters. **(B)** Treatment distribution for Untreated group (NG) and ICB-treating group (EG) in Myeloid cells. **(C)** UMAP plots of annotation for Myeloid clusters. **(D)** The differential number of interactions or interaction strength in the cell-cell communication network **(E, F)** Interaction weights of TAM and TRM clusters under ICB treatment.

To explore the potential roles between myeloid cells and CD8^+^TRM in the TME of HCC, we subclustered the myeloid cells into 12 subtypes (**Figures 5A, B**). After comparing the proportions of them, we found that TAM2, TAM4, TMA5, TAM7 were predominantly after ICB treatment (**Figure 5C**), considering that the influence of TAMs on account of ICB therapy. Additionally, building on our prior characterization of TAM and TRM subsets, we investigated the putative crosstalk between these two cell populations via cellular communication analysis (**Figure 5D**) and found that PD-1^+^TRM cell showed high MIF ligand activity which bound to CD74-CD44 expressed on TAM, while TAMs expressed more LGALS9 interacted with the CD45 which was the ligand on TRM (**Figures 5E, F**).

### 3.7 Distribution of CD8^+^TRM in HCC TME

**Figure 6:**
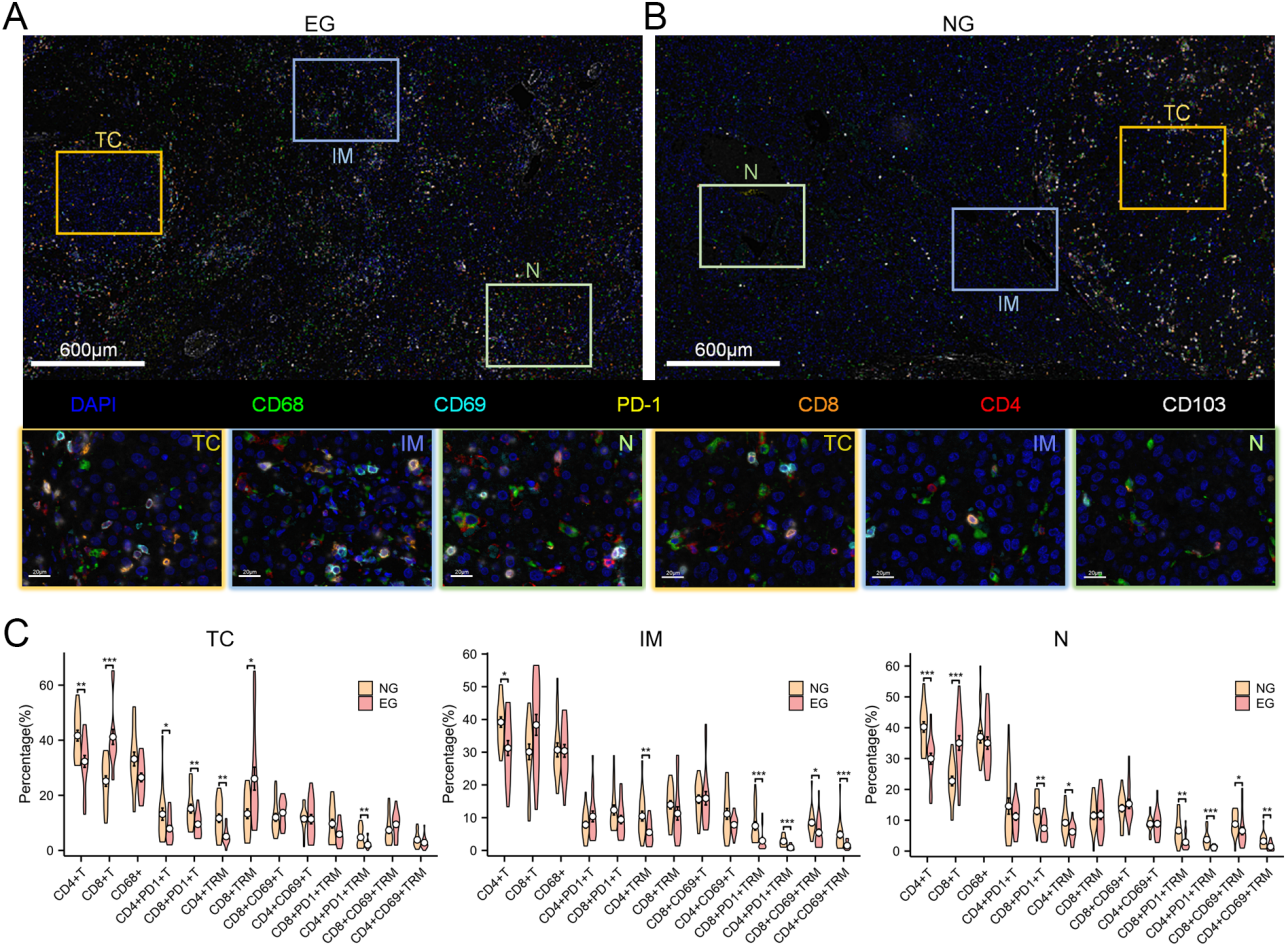
TIICs distribution between different groups. **(A, B)** The acquired immunofluorescence images exhibit the co-localization of corresponding markers and DAPI (nuclear marker). Scale bar: 600 μm and 20 µm. **(C)** Density of TIICs in two groups across the regions of interest. TC, tumor core; IM, invasion margin; N, normal tissue. *P<0.05; **P<0.01; ***P <0.001; Kruskal-Wallis test with Dunn’s multiple comparison test.

To identify the result of scRNA-seq and acquire more details, further investigation of the features of the distribution of TIICs was presented within the HCC tissues (**Figures 6A, B**). We used m-IHC to reveal the spatial density of different TIICs in the TC, IM, and N areas. In the TC and N areas, we found significantly higher total CD8^+^T, lower CD4^+^T cells in ICB-treating group than Untreated group. Compared with the Untreated group, there was a significant increase in CD8^+^TRM cells but a significant decrease in CD4^+^TRM cells in TC area. The proportion of CD8^+^PD-1^+^TRM cells significantly decreased in the N and IM areas, while there was no significant change in the proportion of CD8^+^PD-1^+^TRM cells in the TC area (**Figure 6C**).

### 3.8 Spatial organization of immune cells in HCC with ICB treatment

**Figure 7:**
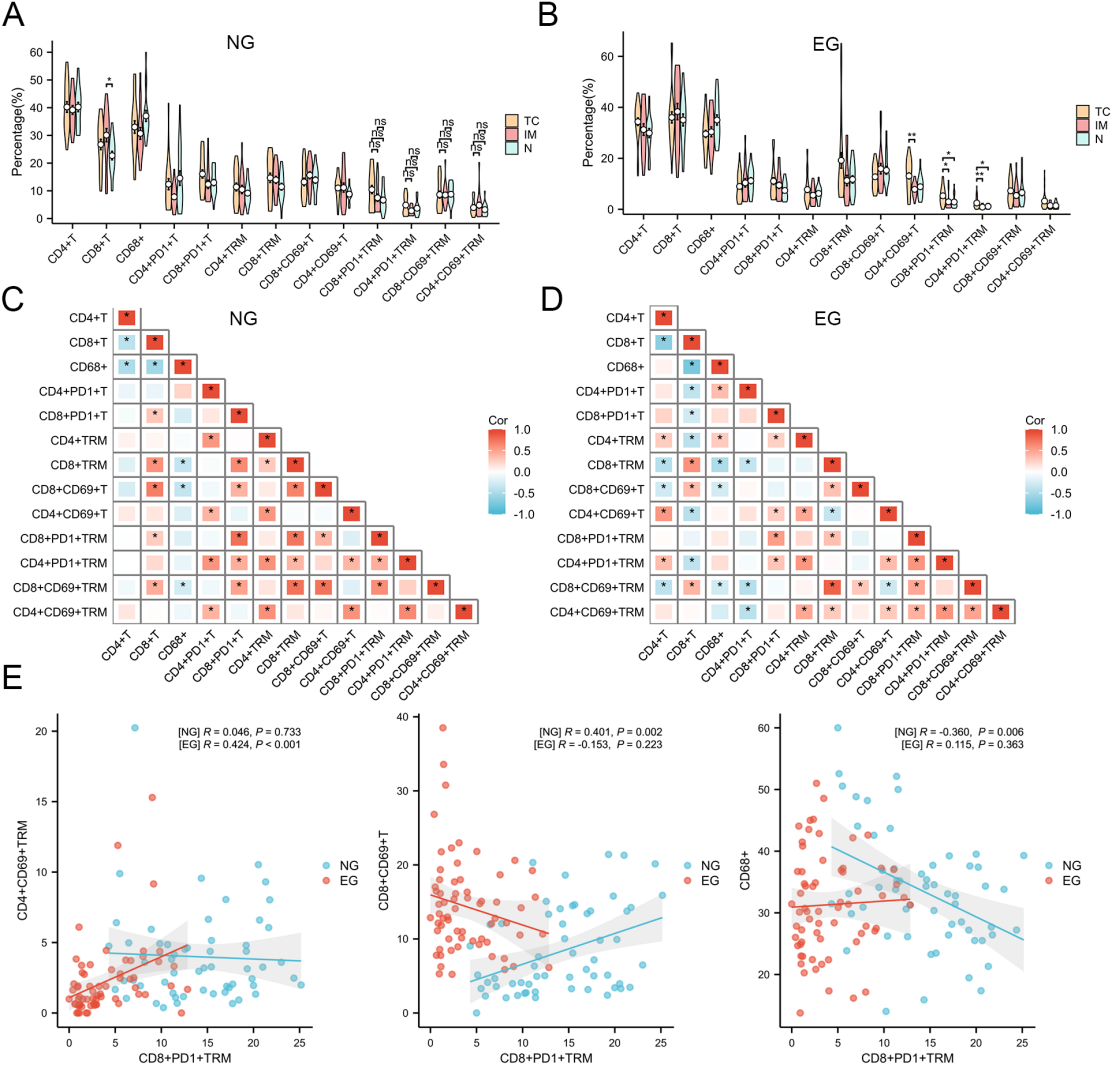
The characteristics of TRM cells in different groups. **(A, B)** Density of TIICs across the regions TC, IM and N in two groups. **(C, D)** The number of interested TIICs were correlated between each other in TC area. **(E)** The correlation between CD8^+^PD-1^+^TRM and different cells (CD4^+^CD69^+^TRM, CD8^+^CD69^+^T, CD68^+^ cells) after ICB treatment. *P<0.05; **P<0.01.

What’s more, we found that there was difference of the distribution of TRM between TC, IM and N in the same group, especially the densities of CD8^+^PD-1^+^TRM and CD4^+^PD-1^+^TRM cells. A significant increase in the overall density of CD8^+^PD-1^+^TRM cells of ICB-treating group was observed within the TC area compared with that in the adjacent tissues; an opposite trend was observed in Untreated group (**Figures 7A, B**). Considering the reason of the heterogeneous distribution of TRM may be the different interactions of each cell have already changed under ICB treatment, we investigated the numerical relationships in the TC region and found that the quantitative correlation in cell numbers between CD8^+^PD-1^+^TRM cells and other immune cells had undergone significant changes (**Figures 7C, D**), the ICB-treating group exhibited a positive correlation between CD8^+^PD-1^+^TRM cells and CD4^+^CD69^+^TRM cells, furthermore, in the untreated group, CD68^+^cells exhibit a negative correlation with CD8^+^PD-1^+^TRM cells and CD8^+^CD69^+^T cells exhibit a positive correlation, whereas in the ICB-treating group, the two cell populations show no significant correlation (**Figure 7E**).

### 3.9 Multi-dimensional TIIC signature after ICB treatment

**Figure 8:**
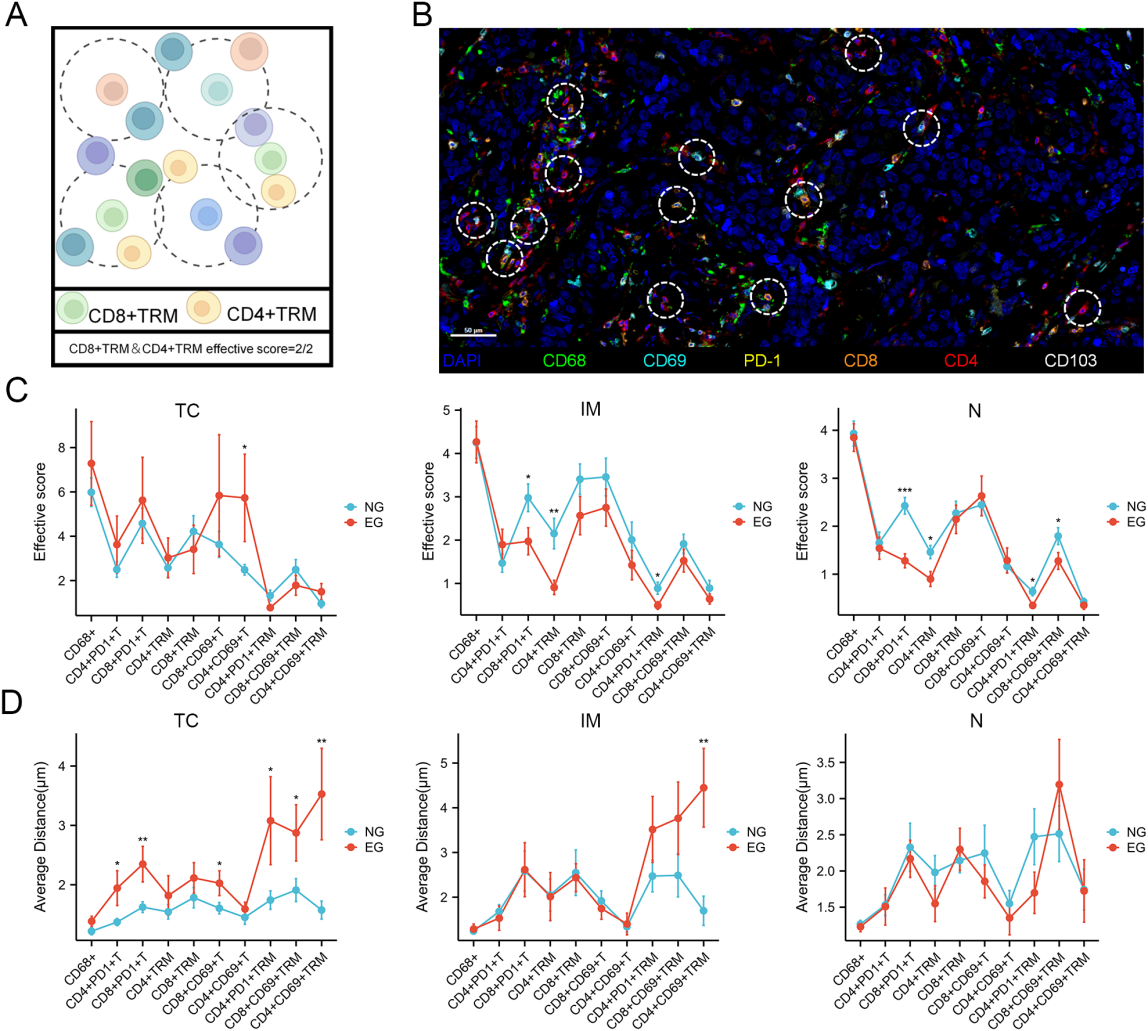
Spatial analysis of CD8^+^PD-1^+^ TRM cells reveals immune cell hierarchical organization. **(A, B)** Diagram of immune cell-focused distance analysis. The white translucent circle represents the radius. Scale bar: 50µm. **(C)** The distribution of the effective score of CD8^+^PD-1^+^TRM cell with other tumor-infiltrating immune cells (TIICs) in the TC, IM and N regions within 30 µm increments. **(D)** The distribution of average distance of CD8^+^PD-1^+^TRM cell to other tumor-infiltrating immune cells in the TC, IM and N regions. TC, tumor core; IM, invasion margin; N, normal tissue. *P<0.05; **P<0.01; ***P <0.001.

To explore the clinical relevance of CD8^+^PD-1^+^TRM cell spatial organization—enabled by our precise single-cell positional mapping—we focused on evaluating the proximity between these cells and other immune populations (**Figures 8A, B**). For quantitative analysis of these interactions, we utilized the pdist bioinformatics function, which computes nucleus-to-nucleus distances across cell types (Methods). To overcome the limitation of analyzing “quantity alone” or “proximity alone,” we developed the “effective score”: a composite parameter that quantifies the fraction of tumor-infiltrating immune cells in close spatial association with CD8^+^PD-1^+^ TRM cells. Specifically, this score was calculated as the number of immune cell-CD8^+^PD-1^+^TRM cells pairs divided by the total number of immune cells across entire tissue slides, thereby preserving spatial variability to a large extent. Using this formula, we found that following immune ICB treatment, the effective score of CD4^+^CD69^+^ T cells in the TC region was significantly higher, whereas the scores of CD8^+^PD-1^+^T cells were lower in IM and N regions (**Figure 8C**). Meanwhile, we calculated the average distance between CD8^+^PD-1^+^TRM cells and each type of immune cell. Under ICB treatment, the distances between CD8^+^PD-1^+^TRM cells and CD8^+^PD-1^+^T cells were reduced in the TC region, while the distance between CD8^+^PD-1^+^TRM cells and CD4^+^CD69^+^T cells was increased in the IM region (**Figure 8D**). Meanwhile, we also observed that CD8^+^PD-1^+^TRM cells and CD68^+^ cells had the highest effective score and the shortest average distance, indicating the closest association between these two cell types, which is consistent with the previous cellular communication analysis results. Combined with the results of correlation analyses between TIICs and CD8^+^PD-1^+^TRM cells, as well as intercellular communication assays, we propose that the changes of CD8^+^PD-1^+^TRM cells distribution following ICB treatment may be associated with interactions between CD8^+^PD-1^+^T cells and CD68^+^ cells.

## 4 Discussion

ICB treatment, via its capacity for reinvigorating immune cells to exert anti-tumor effects, offers a novel, promising approach for improving survival outcomes of cancer patients. Following ICB, the state of CD8^+^ T cells, both within and outside the TME, possesses a differential capacity to respond, mobilize and seed effective antitumor immune responses at these sites (39), In addition, there is proliferation and activation of Treg cells within the tumor, as well as tumor-draining lymph nodes and its circulation (40) along with an increased number of anti-inflammatory macrophages which can then induce an increase in CCL5 (41).

TRM cells are dynamically generated both within the tumor microenvironment and across extrapulmonary tissue compartments in response to tumor progression and therapeutic perturbations. Notably, PD-1/PD-L1-blocking antibodies exert both direct and indirect effects to potently augment multiple functional attributes of CD8^+^ TRM cells due to they express a variety of immune checkpoint molecules, including PD-1, CTLA-4, Lag-3, and Tim-3, and the expression levels of these molecules are closely associated with the exhaustion status of TRM cells (42). With robust evidence from preclinical and clinical studies: Three such examples include: 1) Ex vivo restimulation with anti-PD-1 was found to restore the proliferation and cytokine production of CD39^+^CD8^+^ TRM cells derived from triple-negative breast cancer (TNBC) (43), 2) *In vivo* anti-PD-L1 treatment markedly boosted cytokine secretion by gastric cancer-derived CD8^+^TRM cells (44), 3) Dual checkpoint blockade (anti-PD-1/CTLA-4) strengthened the expansion and cytotoxic potential of CD8^+^TRM-like cells (45).

In this study, we established a robust multistep multi-spectral imaging platform tailored for HCC tissues, characterized by its ability to generate high-fidelity, single-cell resolution datasets. Leveraging this technology, we achieved two critical objectives: (1) quantitative profiling and spatial mapping of TRM cell subsets with heterogeneous phenotypes such as CD8^+^TRM cells; and (2) assessment of these cellular and spatial parameters as candidate biomarkers. This work is particularly timely given the clinical context of HCC immunotherapy: while immune checkpoint inhibitors have revolutionized treatment paradigms, their effectiveness is restricted to a minority of patients, and current tools fail to reliably predict responders (46, 47). Responses to ICB have been associated with TIICs, an increase in tumor-infiltrating PD-1^hi^CD8^+^T cells, LAG3^+^CD8^+^T cells and monocytes/macrophages in tumor as well as S100A9^+^CD14^+^monocytes in the peripheral blood of patients exhibiting suboptimal responses to anti-PD-1 therapy (48–50). Therefore, infiltration of TIICs can be a prognostic factor for ICB responses. However, most research currently conducted in this area has focused on the density of certain TIICs, while ignoring their spatial distributions.

Our current results reveal that the proportions of CD8^+^T and CD8^+^TRM cells were increased in HCC tissues of patients with ICB treatment, and the interaction between TRM and CD68^+^cells was enhanced. Another significant outcome from our study was the finding that the distribution of CD8^+^PD-1^+^TRM cells was enhanced in the TC region of the ICB-treating group, while in the untreated group, CD8^+^ PD-1^+^TRM cells showed homogeneous infiltration across the three regions. The proportion of CD8^+^PD-1^+^ TRM cells did not change significantly from N to IM to TC in the untreated group, while in the ICB-treating group, the proportion of CD8^+^PD-1^+^TRM cells increased significantly from the N to IM and then to the TC region. The potential mechanisms for the enhanced infiltration of TRM cells may be that ICB treatment increases the secretion of CXCL9/CXCL10 by tumor cells, which binds to CXCR3 on the surface of CD8^+^TRM cells to recruit and promote their infiltration into the TC region; alternatively, ICB treatment induces TAMs to secrete IL-12, which promotes the differentiation of CD8+T cells into TRM cells and enhances their infiltration ability (51, 52). It has been recently reported that CD8^+^TRM possess potent tumor-killing capabilities and can promote the colocalization of T cells with B cells and dendritic cells, and the accumulation in TC shows a significant correlation with the efficacy of ICB (53, 54).

Patients treated with ICB achieved PR or SD in our research, may reflect the positive effects of ICB treatment in our center, and associated with these beneficial effects was an accumulation of CD8^+^TRM cells in TC region and CD8^+^PD-1^+^TRM cells toward the HCC center. A preliminarily conclusion from these findings is that ICB treatment in HCC produces an infiltration of CD8^+^TRM cells into TC region and increases the number of CD8^+^PD-1^+^TRM cells from the N to TC region. In support of this conclusion are the findings which demonstrate positive effects of CD8^+^PD-1^+^TRM cells after ICB treatment (55, 56), but these CD8^+^PD-1^+^TRM cells may be related to a poor prognosis in pancreatic ductal adenocarcinoma and glioblastoma without ICB treatment (57, 58). In this way, the function of CD8^+^PD-1^+^TRM cell may undergo a subversive change in their function from tumor promotion to tumor suppression in response to ICB treatment. Moreover, increased numbers of such cells in the ICB-treating group may exert stronger antitumor effects as observed in our study.

Radiomics analysis is defined as a computer-assisted data mining approach that extracts quantitative high-throughput features from medical images. The quantitative analysis of CD8^+^T cells usually depends on pathology, however, by delineating the tumor three-dimensional and extracting radiomics features, the degree of CD8^+^T cell infiltration can be observed, and then the efficacy of immunotherapy can be predicted (59). The GLSZM-derived Small Area High Gray Level Emphasis and wavelet_LHH_glcm_Imc2 are two features focusing on small, high-intensity areas, which may reflect the microstructure inside the tumor, such as cell density. However, it should be noted that our radiomic model was constructed based on a small sample cohort (n=50) with a limited validation set (n=10). The wide confidence interval of the area under the curve (AUC = 0.308–1.000) indicates insufficient model stability. This limitation may restrict the reliability of the model in clinical practice: the small sample size and limited validation could introduce potential selection bias, reduce the generalizability of the model to larger and more diverse patient populations, and thus affect the accuracy of guiding clinical decision-making. We can jointly establish an immune cell infiltration model by integrating radiomics and bioinformatics analysis results to predict the efficacy of ICB therapy (60, 61).

One of the strengths of this study lies in utilizing radiomics technology and multi-omics analyses to systematically evaluate the entire TME of patients. Such an acquisition permits a continuous and comprehensive comparison of the distribution of CD8^+^ T cells and further extends to CD8^+^ TRM cells, offering a window into the adaptive remodeling of the tumor microenvironment under immunotherapy. A notable limitation was our inability to obtain a more accurate survival analysis due to the lack of patient prognostic information. Accordingly, further large cohort studies, ideally with prospective clinical trials, will be required to substantiate our findings.

## Data Availability

The datasets presented in this study can be found in online repositories. The names of the repository/repositories and accession number(s) can be found in the article/**Supplementary Material**.

## Supplementary material

Supplementary Figure 1 Cohort overview.

Supplementary Table 1 Antibody Information Used in Experiments.
