# Supplementary figures and images for: Multi-omics spatial characteristics of CD8+TRM cells in hepatocellular carcinoma and immunotherapy response prediction
Source: Front Immunol. 2025 Dec 8;16:1710741. doi: 10.3389/fimmu.2025.1710741 (PMC12719432; doi:10.3389/fimmu.2025.1710741)

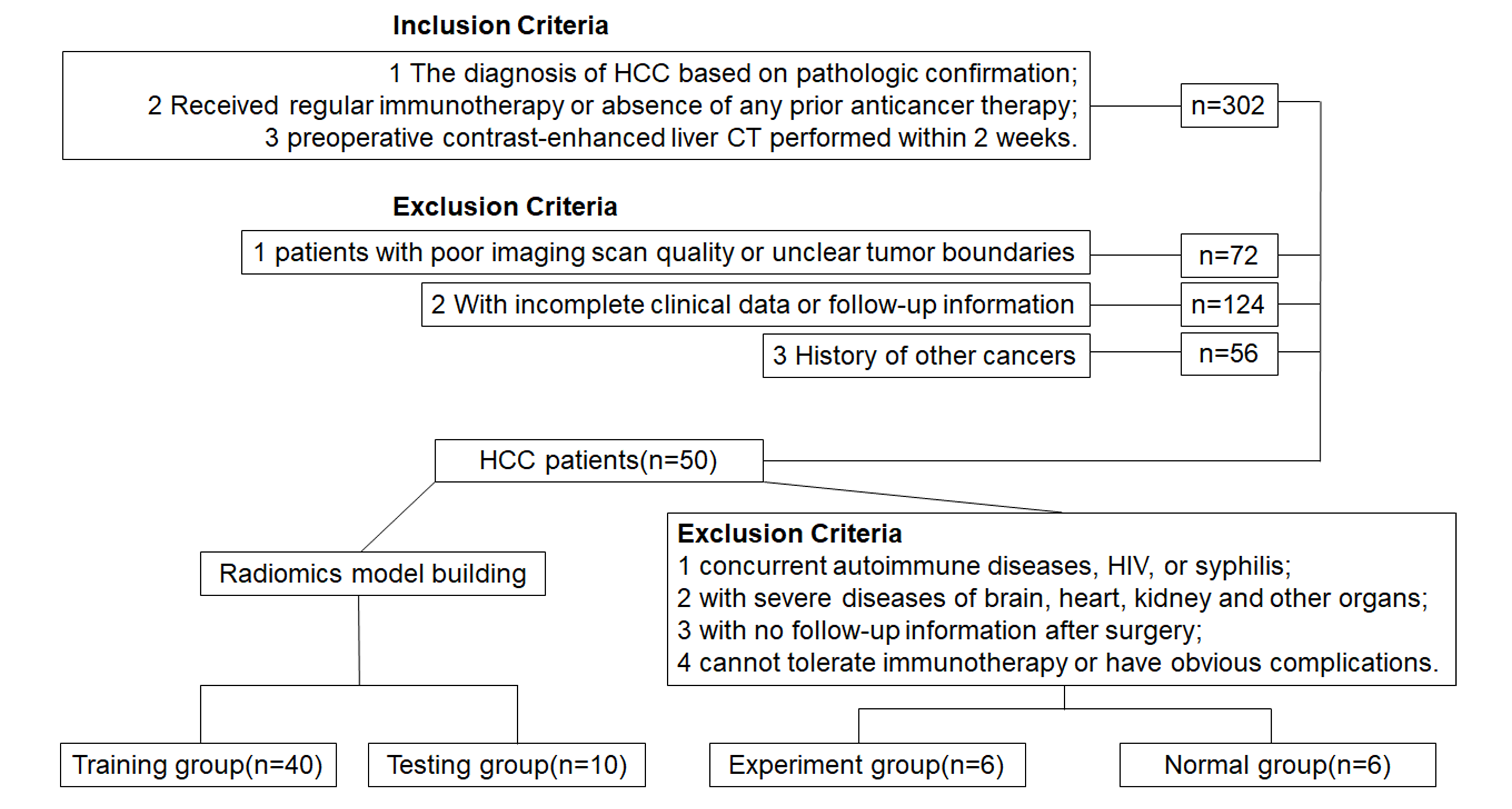

Supplement: Supplementary Figure 1 — Cohort overview. [file Image1.tif]
