# Supplementary material for: Multi-omics spatial characteristics of CD8+TRM cells in hepatocellular carcinoma and immunotherapy response prediction
Source: Front Immunol. 2025 Dec 8;16:1710741. doi: 10.3389/fimmu.2025.1710741 (PMC12719432; doi:10.3389/fimmu.2025.1710741)
Supplement: Supplementary Table 1 — Antibody Information Used in Experiments. [file Table1.docx]

| Antibody | Supplier | Dilution | Clone | Catalog |
| --- | --- | --- | --- | --- |
| CD4 | Abcam | 1:150 | EPR6855 | ab133616 |
| CD8 | Abcam | 1:150 | CAL66 | ab237709 |
| CD68 | Abcam | 1:100 | EPR20545 | ab213363 |
| CD69 | Abcam | 1:150 | RM1274 | ab322534 |
| CD103 | Abcam | 1:100 | EPR4166(2) | ab271889 |
| PD1 | Abcam | 1:100 | CAL20 | ab237728 |
